# Editorial: Working and absence from work during the pandemic

**DOI:** 10.3389/fpsyg.2024.1411403

**Published:** 2024-06-17

**Authors:** Hana Brborović, Dragan Mijakoski, Milan Milošević, Ognjen Brborović

**Affiliations:** ^1^Department of Environmental and Occupational Health and Sports Medicine, Andrija Štampar School of Public Health, School of Medicine, University of Zagreb, Zagreb, Croatia; ^2^WHO Collaborating Centre, GA2LEN - Global Allergy and Asthma Excellence Network Collaborating Centre, Institute of Occupational Health of RN Macedonia, Skopje, North Macedonia; ^3^Faculty of Medicine, Ss.Cyril and Methodius, University in Skopje, Skopje, North Macedonia; ^4^Department of Social Medicine and Organization of Health Care, Andrija Štampar School of Public Health, School of Medicine, University of Zagreb, Zagreb, Croatia

**Keywords:** absenteeism, presenteeism, pandemic (COVID-19), occupational medicine, workers, wellbeing, engagement

The Pandemic has taught us new lessons that needed to be learned many years ago: absence from work is not always an absence. Some people are highly engaged in work tasks when they work from home, while others reach their maximum potential working from the office. Some, like front-line workers, including health workers, have no other option but to work at their workplace. During the pandemic we have learned that coming to work while sick (sickness presenteeism) is not a noble thing to do, but represented a threat to themselves and others. Staying at home while sick (sickness absenteeism), has become a noble and mandatory act.

In this Research Topic, we have asked the researchers for a deeper insight and understanding from both workers' and/or employers' perspective about the absence from work and working life in general, during the pandemic. We have laid down numerous questions. What level of engagement did employees have during the pandemic? How did this new context influence the health and wellbeing of workers, as well as economic processes? Furthermore, we were very interested in the “user experience.” Did the “new normal” come with a price? Was the new normal at least normal? Should we remain in the new normal once the pandemic is over or should we go back to the old?

After one and a half years of collection and review, out of 34 submitted papers, 19 have reached the publication decision. Two out of the 19 accepted papers are mini-reviews, while 17 are original research papers, including 141 co-authors altogether. Each of these 19 seminal papers dissects unique dimensions of the global health crisis during COVID-19, ranging from psychological impacts on workers and shifts in healthcare systems to remote work, stress, adaptation and coping, organizational communication, and eventually presenteeism. These studies offer critical insights into the multifaceted consequences and adaptive strategies used during this period.

Several studies, including those by Kröner and Müller, Milaković et al., and Orešković et al., touch upon the shift to remote work during the pandemic. These studies underline the impact of telework on musculoskeletal disorders, job satisfaction, work-life balance, and mental wellbeing, highlighting both the benefits and challenges associated with remote work arrangements. The study by Milaković et al. critically examines the emerging trend of telework and its association with musculoskeletal disorders (MSDs). Highlighting the importance of ergonomic setups and balanced work-life boundaries, this research provides essential guidance on how to navigate the challenges of remote work environments. Orešković et al. scrutinize the association between remote work and job satisfaction. Their large-scale survey counters prior assumptions about remote work's disadvantages, advocating for employee-centric work arrangement policies. Kröner and Müller investigate the longitudinal impact of telework on mental wellbeing. Their identification of three distinct wellbeing trajectories among German workers reveals telework as a potential buffer against work-related stressors, offering valuable insights for future remote work policies.

The pandemic-induced stress and mental health issues are a recurring theme. Studies such as those by Jia et al., Koren et al., and Tsubono and Mitoku emphasize the increased levels of stress among various professional groups, including teachers, healthcare workers, and general employees. These studies highlight the importance of addressing mental health and stress management in these challenging times. Research by Tsubono and Mitoku delves into the occupational stress of public school teachers in Japan during the pandemic. The nationwide survey pointed out the overarching issues of quantitative workload and long working hours, along with school-type-specific stressors such as managing extracurricular activities in junior high schools. This study is significant for its comprehensive approach to understanding the various stress factors in education and the importance of addressing them through tailored strategies. The 2023 study by Jia et al. focuses on the physical discomforts experienced by 515 front-line medical personnel in China. In particular, the study brings to light severe physical discomforts, including dyspnea and pain, primarily attributed to the prolonged use of personal protective equipment (PPE). This underscores the pressing need to address the physical and ergonomic aspects of medical work during health crises. Koren et al. review the psychosocial risks emerging from the pandemic and their impacts on mental health. They highlight crucial factors such as isolation, job insecurity, and digitalization-induced stress, emphasizing the need for workplace mental health interventions. This study is critical for understanding the broad spectrum of pandemic-related stressors and their implications for mental health.

Papers by Mijakoski et al., Schmidt-Stiedenroth et al., and Veje et al. discuss the resilience and challenges faced by healthcare systems and professionals. These studies explore how the pandemic has strained healthcare workers, highlighting the need for supportive measures and organizational support to maintain their wellbeing and effectiveness. Mijakoski et al. conducted a comprehensive analysis of the relationship between burnout and job demands and resources among healthcare workers in Southeast European countries. With 4,621 HWs, the research used the Maslach burnout inventory and other tools to assess burnout dimensions, job demands, and resources such as remuneration and supervisory relationships. Significant differences in emotional exhaustion were found between countries and in job demands/resources. The findings indicate that job demands and resources predict emotional exhaustion. The study concludes that preventive measures for the mental health of HWs should consider country-specific contexts and prepare for future crises. Schmidt-Stiedenroth et al.'s investigation into the psychosocial stressors and resources among hospital staff in Germany provides crucial insights into crisis management within healthcare institutions. Their research, based on 303 responses, underscores the importance of psychosocial support, clear communication, and efficient workload management to maintain the wellbeing of healthcare professionals during crises. In the study by Veje et al., the experiences of healthcare workers in a Swedish university hospital's infectious disease department during the pandemic are meticulously analyzed. The research emphasizes the increased workload and emotional stress, advocating for well-structured support systems for healthcare workers, especially younger employees and those with greater concerns about infection.

Adaptation to the challenges of the pandemic is a key focus of studies like those of Härgestam et al., Straßburger et al. and Tien et al.. These studies illustrate how professionals, including physicians and female STEM workers, adapted and coped with the new realities of the pandemic, highlighting resilience and flexibility in face of adversity.

Härgestam et al. provide a compelling narrative on the challenges Swedish physicians faced in preserving their professional identity amid the pandemic. Their research reveals the tensions between traditional medical practice and the exigencies of a public health crisis, shedding light on the adaptability and ethical quandaries encountered by medical professionals. Tien et al. provide an insightful look at the coping strategies of female STEM professionals in Silicon Valley during the pandemic. Their in-depth interviews reveal how these women effectively navigated new work-life challenges, emphasizing the value of flexibility and support systems. Straßburger et al. explore the return-to-work process for employees affected by Post-COVID syndrome. Their focus on personal coping resources and organizational offerings highlights the necessity for a multidisciplinary approach in facilitating reintegration.

The importance of organizational communication and support is evident in studies by Björk et al., Côté et al., and Ibañez et al.. These papers shed light on the role of effective communication and organizational strategies in managing pandemic-related challenges in workplaces, emphasizing the need for clear, consistent, and culturally sensitive messaging. The 2024 study by Côté et al. critically examines communication barriers and strategies in Quebec and Ontario workplaces during the pandemic. The study reveals the challenges faced by essential workers in precarious employment and underscores the importance of effective and adaptable communication in crisis management. In the study by Ibañez et al., the resilience of family businesses in Chile during the pandemic is highlighted. Surveying 516 employees, the study found that these businesses effectively counteracted burnout and bolstered affective commitment, thereby elevating job satisfaction during challenging times. This research is crucial as it shines a light on adaptive capabilities and the unique work culture of family firms, underlining the importance of nurturing employee commitment as a buffer during crises. Björk et al. examine the challenges faced by healthcare managers in Sweden during the first wave of COVID-19. Their mixed-method approach highlights the critical need for clear communication and adequate support for managers to adapt to rapid changes and maintain workforce wellbeing.

Finally, the theme of presenteeism is explored in the study by Biron et al.. It examines how presenteeism, as an adaptive behavior, is influenced by the work environment and health limitations, thus impacting performance. This highlights the complexity of presenteeism and the need for nuanced workplace health management. Biron et al. offer an innovative perspective on presenteeism, categorizing it into functional, dysfunctional, overachieving, and average types. This study is instrumental in understanding the complex interaction between health and performance on the job, which calls for tailored interventions.

These studies collectively offer a multifaceted view of the challenges and adaptations during the COVID-19 pandemic in different sectors. They reveal the crucial role of organizational support, communication strategies, and individual resilience in navigating the complexities of the pandemic. This Research Topic not only documents immediate responses to the crisis, but also provides valuable insight for future preparedness in similar global challenges. In conclusion, the COVID-19 pandemic has led to a shift in work patterns. These changes have been influenced by various factors including health behaviors, work-related factors, and the blurring of boundaries between work and home.

We express our profound gratitude to all the authors who recognized the significance of our theme and contributed their valuable work to this Research Topic. Your insightful papers have not only enriched this Research Topic, but have also significantly advanced our understanding of the multifaceted impacts of the COVID-19 pandemic. Our heartfelt thanks go to all the reviewers who dedicated their time and expertise to meticulously assess each submission with their constructive feedback and rigorous evaluations have been indispensable in upholding the quality and integrity of this publication. The authors are also deeply grateful to Frontiers in Psychology for their collaboration and trust in this endeavor. The support and guidance provided by the journal team have been crucial in bringing this Research Topic to fruition. This collaborative effort has resulted in a Research Topic that we believe will be a valuable resource for readers and researchers alike. We thank you all for your contributions, dedication, and commitment to advancing scientific knowledge in these challenging times.

## Author contributions

HB: Conceptualization, Resources, Supervision, Validation, Writing – original draft, Writing – review & editing. DM: Conceptualization, Resources, Supervision, Validation, Writing – original draft, Writing – review & editing. MM: Conceptualization, Resources, Supervision, Validation, Writing – original draft, Writing – review & editing. OB: Conceptualization, Resources, Supervision, Validation, Writing – original draft, Writing – review & editing.

